# Reliability and validity of manual palpation for the assessment of patients with low back pain: a systematic and critical review

**DOI:** 10.1186/s12998-021-00384-3

**Published:** 2021-08-26

**Authors:** Paul S. Nolet, Hainan Yu, Pierre Côté, Anne-Laure Meyer, Vicki L. Kristman, Deborah Sutton, Kent Murnaghan, Nadège Lemeunier

**Affiliations:** 1 Department of Graduate Education and Research, Canadian Memorial Chiropractic College, Toronto, Ontario Canada; 2 School of Kinesiology, Lakehead University, Thunder Bay, Ontario Canada; 3 CAPHRI School for Public Health and Primary Care, Faculty of Health, Medicine, and Life Sciences, Maastricht University, 6211 LM Maastricht, The Netherlands; 4 Institute for Disability and Rehabilitation Research, Ontario Tech University, Oshawa, Ontario Canada; 5 Faculty of Health Sciences, University of Ontario Institute of Technology (UOIT), Oshawa, Ontario Canada; 6 Institut Franco-Européen de Chiropraxie, Toulouse, France; 7 EPID@Work Research Institute, Department of Health Sciences, and the Division of Human Sciences, Northern Ontario School of Medicine, Lakehead University, Thunder Bay, Ontario Canada; 8 Institute for Work and Health, Toronto, Ontario Canada; 9 Canadian Memorial Chiropractic College, Toronto, Ontario Canada; 10 UMR1295, Université de Toulouse, UPS, Inserm, Toulouse, France

**Keywords:** Manual palpation, Reliability, Validity, Assessment, Low back pain, Systematic review

## Abstract

**Abstract:**

**Background:**

Static or motion manual palpation of the low back is commonly used to assess pain location and reproduction in low back pain (LBP) patients. The purpose of this study is to review the reliability and validity of manual palpation used for the assessment of LBP in adults.

**Method:**

We systematically searched five databases from 2000 to 2019. We critically appraised internal validity of studies using QAREL and QUADAS-2 instruments. We stratified results using best-evidence synthesis. Validity studies were classified according to Sackett and Haynes.

**Results:**

We identified 2023 eligible articles, of which 14 were low risk of bias. Evidence suggests that reliability of soft tissue structures palpation is inconsistent, and reliability of bony structures and joint mobility palpation is poor. We found preliminary evidence that gluteal muscle palpation for tenderness may be valid in differentiating LBP patients with and without radiculopathy.

**Conclusion:**

Reliability of manual palpation tests in the assessment of LBP patients varies greatly. This is problematic because these tests are commonly used by manual therapists and clinicians. Little is known about the validity of these tests; therefore, their clinical utility is uncertain. High quality validity studies are needed to inform the clinical use of manual palpation tests.

**Supplementary Information:**

The online version contains supplementary material available at 10.1186/s12998-021-00384-3.

## Introduction

Low back pain (LBP) is the most prevalent musculoskeletal condition in the general population [1, 2]. The point prevalence of LBP ranges between 1 to 58.1% and one-year prevalence ranges between 0.8 to 82.5% [3] depending of the LBP definition and population. LBP is the leading cause of years lived with disability and is the sixth leading cause of disability adjusted life years globally [4, 5] and it is associated with poor health-related quality of life and has a substantial economic burden to society [6, 7]. Non-specific LBP is more common than specific LBP (e.g., cancer, fractures, infectious disorders, or ankylosing spondylitis) and it cannot be attributed to a specific underlying pathology [8].

The clinical assessment of low back pain involves completing a physical examination [9]. Manual palpation is a common tool used to assess patients with LBP [10]. It includes static and dynamic palpation of soft tissue or joints and aims to identify painful structures and biomechanical dysfunction of the spine [11]. However, the clinical utility of these tests is controversial.

Previous systematic reviews have investigated the reliability and validity of manual palpation for the assessment of patients with LBP [9, 11–13]. According to these reviews, the inter-rater reliability of static joint and soft-tissue palpation to locate pain is poor (kappa (k) ≤ 0.40), and the inter-rater reliability of static palpation for soft tissue changes (e.g., tension) is inconsistent [9, 11, 13]. Furthermore, one review reported that motion palpation may be valid in detecting decreased motion, or lack of end-play in the lumbar spine [12]. However, motion palpation may not be valid to detect aberrant motion of the sacroiliac joints [12]. These reviews are outdated and there is a need for an up-to-date systematic review. The purpose of our systematic review was to determine the reliability and validity of manual palpation used to assess adult patients with LBP.

## Methods

### Eligibility criteria

#### Population

We included studies of adults (≥18 years) with LBP. LBP refers to pain or discomfort below the costal margin and above the inferior gluteal folds and can be with or without referred leg pain [14]. Our systematic review includes patients with non-radicular low back pain, radicular low back pain, spinal stenosis, degenerative or isthmic spondylolisthesis, and failed back surgery syndrome.

#### Definitions

Our review focuses on studies assessing the reliability or validity of manual palpation for the assessment of patients with LBP. Reliability describes the consistency of measurements across people or instruments [15]. Validity is the degree to which a test measures what it is intended to measure [15].

Manual palpation is a diagnostic procedure where the examiner feels with their hands to assess the mobility and state of the soft and boney tissues [16]. Palpation techniques include both static and dynamic (motion) methods, which are often used to identify areas of tissue pain and dysfunction, target manual and manipulative therapies and determine effectiveness of the intervention [9]. Static palpation is used to identify bony asymmetry of bony landmarks, tender points, and trigger points to evaluate tissue texture, temperature and tone [17]. Motion palpation is used to assess the quantity and quality of movement through the lumbar spine and pelvis [17]. Motion palpation assessment can be continuous within the normal range of motion with joint play, or dynamic soft tissue palpation or end range assessment for end-feel or joint springing [17]. Palpation involving devices such as pressure algometry were excluded.

#### Outcomes

We aimed to evaluate clinical outcomes assessed by palpation. Outcomes include pain, segmental mobility and stiffness for static joint palpation; joint movement and position assessed for motion joint palpation; and pain, tenderness, trigger points, muscle contraction assessed for static soft tissue palpation.

#### Study characteristics

Eligible studies met the following inclusion: 1) English or French language; 2) published in peer reviewed journals between January 1, 2000 to July 11, 2019; 3) assessing the reliability or validity of manual palpation. Previously published systematic reviews on this topic were included in our review. Comparing our systematic review with previous systematic reviews examined findings of studies published before 2000. We excluded: 1) letters, guidelines, editorials, commentaries, unpublished manuscripts, dissertations, reports, book chapters, conference proceedings and abstracts, lectures, addresses, and consensus statements; 2) cadaveric and animal studies; 3) literature reviews and case studies; 4) studies targeting individuals with serious pathology (e.g., fractures, dislocations, systemic disease, myelopathy, neoplasm and infection; and 5) studies with sample size < 20 per group.

### Search strategy and data sources

The search strategy was developed in consultation with a health sciences librarian and a second librarian was consulted to ensure accuracy and completeness using the Peer Review of Electronic Search Strategies PRESS checklist [18]. We systematically searched the following electronic databases: MEDLINE, CINAHL, PubMed, Cochrane Central Register of Controlled Trials, and SPORTDiscus. Search terms consisted of subject headings specific to each database (e.g. MeSH in MEDLINE) and free text words relevant to LBP, diagnosis, reliability, validity, and palpation (Additional file 1).

### Study selection

Identified citations were exported into EndNote for reference management and tracking of the screening process. We screened articles in two stages. In stage one, titles and abstracts were screened for their relevance by pairs of independent reviewers (NL, PN, ALM). Stage two involved screening the full text article of all possibly relevant citations from stage one. Disagreements on screening stages were discussed between reviewers to reach consensus. When consensus could not be reached, a third reviewer independently screened the citation and discussed with the two reviewers to reach consensus.

### Assessment of risk of Bias

Three reviewers (NL, PN, ALM) critically appraised all relevant studies (Tables 1 and 2) using the modified Quality Appraisal Tool for Studies of Diagnostic Reliability (QAREL) [33] criteria to assess the internal validity of the diagnostic reliability studies and the modified Quality Assessment of Diagnostic Accuracy Studies-2 (QUADAS-2) [34] criteria to assess diagnostic accuracy/validity studies (Additional files 2 and 3). The original QAREL and QUADAS-2 instruments were modified to include: 1) not applicable options; 2) a question regarding the clarity of the study objective; and 3) the *Sackett and Haynes* classification (phases of validity studies in QUADAS-2 instrument). If a study was judged as “low” on all domains relating to bias or applicability then it was appropriate to have an overall judgment of “low risk of bias” or “low concern regarding applicability” for that study. If a study was judged “high” or “unclear” on one or more domains then it may be judged “at risk of bias” or as having “concerns regarding applicability” [33, 34]. We included low risk of bias studies in our best evidence synthesis.

**Table 1:** Risk of bias for scientifically admissible reliability studies based on the modified QAREL criteria

Author, Year	Study objective clearly described	Representative sample	Representative raters	Blinded to others’ findings	Blinded to own findings	Blinded to results of ref std	Blinded to clinical information	Blinded to additional clues	Order of exam varied	Appropriate time interval	Test applied correctly	Appropriate statistical measures
Alyazedi et al. 2015 [19]	Y	Y	Y	Y	N/A	N/A	Y	Y	UC	Y	Y	Y
Arab et al., 2009 [20]	Y	Y	Y	Y	N	N/A	Y	Y	Y	Y	Y	Y
Downey et al., 2003 [21]	Y	Y	Y	Y	N/A	N/A	UC	UC	Y	Y	Y	Y
Hebert et al., 2015 [22]	Y	Y	Y	Y	N/A	Y	UC	UC	Y	Y	Y	Y
Hicks et al. 2003 [23]	Y	Y	Y	Y	N/A	N/A	UC	UC	UC	Y	Y	Y
Jensen et al. 2013 [24]	Y	Y	Y	Y	N	N/A	UC	UC	Y	Y	Y	Y
Ravenna et al., 2011 [25]	Y	Y	Y	Y	N/A	N/A	N	UC	Y	Y	Y	Y
Schneider et al., 2008 [26]	Y	Y	Y	Y	N/A	N/A	Y	Y	Y	Y	Y	Y
Tong et al., 2006 [27]	Y	Y	Y	Y	N/A	N/A	N	UC	UC	UC	Y	Y
Walsh et al., 2009 [28]	Y	Y	Y	Y	N/A	Y	N	UC	N	Y	Y	Y
Weiner et al., 2006 [29]	Y	Y	Y	Y	N/A	UC	Y	UC	UC	Y	Y	Y

*N* No, *N/A* Not applicable, *ref. std.* reference standard, *UC* Unclear, *Y* Yes

**Table 2:** Risk of bias for scientifically admissible validity studies based on the modified QUADAS-2 criteria

Author, Year	Consecutive sample	Case-control design avoided	Avoided inappropriate exclusions	Blinded to ref std results	Pre-specified threshold	Described ref std	Appropriate ref std	Blinded to index test results	Appropriate time interval	All patients received ref std	All patients received same ref std	All patients included in analysis
Adelmanesh et al., 2016 [30]	Y	Y	Y	Y	N/A	Y	Y	Y	Y	Y	Y	Y
Hebert et al., 2015 [22]	N	Y	Y	Y	N/A	Y	Y	Y	Y	Y	Y	UC
Koppenhaver et al., 2013 [31]	N	Y	Y	Y	N/A	Y	Y	Y	UC	Y	Y	Y
Soleimanifar et al., 2017 [32]	N	Y	Y	N	Y	Y	Y	N	Y	Y	Y	Y
Walsh et al., 2009 [28]	Y	Y	Y	Y	N/A	Y	UC	Y	UC	Y	Y	Y
Weiner et al., 2006 [29]	N	N	Y	N/A	N/A	N/A	N/A	N/A	N/A	N/A	N/A	UC

*N* No, *N/A* Not applicable, *ref. std.* reference standard, *UC* Unclear, *Y* Yes

Validity studies with low risk of bias were classified into one for four phases of investigation following the recommendation of Sackett and Haynes [35]. The purpose of phase I studies is to determine if test results are different for LBP patients and healthy controls. The purpose of Phase I studies is to determine whether test results differ between LBP patients and healthy controls. This information is useful to justify Phase II studies. Phase II studies aim to determine whether patients with a positive palpation result are more likely to have decreased functions, severe disability or structure changes (e.g., spinal stenosis) than patients with a negative result. Phase I and II studies provide preliminary evidence that a test should to be tested in phase III studies. On their own, results from phase I and II studies cannot be used to confirm the validity of tests. However, according to Sackett and Haynes classification, phase I – II justify that a test should be further investigated. Phase III studies aim to determine whether a test result can distinguish between LBP patients with suspected conditions (e.g., radiculopathy). Finally, Phase IV studies aim to determine whether patients who undergo a manual palpation test have a better prognosis than similar patients who were not tested [35]. Phase IV studies are a unique type of studies that differ from phase I-III studies in examining diagnostic accuracy. Low risk of bias of phase IV study would be assessed using the Scottish Intercollegiate Guidelines Network (SIGN) criteria [36].

### Data extraction and synthesis of results

One reviewer (PN) extracted data from low risk of bias studies and built evidence tables (Tables 3 and 4); and two reviewers (NL or HY) verified the accuracy and completeness of the data extraction. The reliability and validity studies were stratified according to targeted body structures (joint or soft tissue), technique (static or motion palpation), and clinical outcome (pain provocation, mobility, or stiffness). We used qualitative synthesis to synthesize the best evidence [37]. Eligible statistics include 1) means, median and/or percent in phase I studies; 2) correlations, sensitivity, specificity, positive predictive value, negative predictive value and/or likelihood ratio in phase II or III studies; and 3) prevalence in phase III studies.

**Table 3:** Evidence table for low risk of bias studies assessing the reliability of manual palpation tests in patients with low back pain

Authors, Year Country	Design Sample Size (n)	Case Definition	Index Test	Reliability
***Static Joint Palpation (n = 262)***				
Alyazedi et al. 2015 [19] USA	Inter-rater reliability (*n* = 40)	Recurrent LBP or chronic LBP (≥ 3 months), 21–71 yrs. old.	Prone instability test was done in two parts: 1) relaxation phase: the subject was lying prone on the examination table with feet on the floor. The examiner performed PA mobility testing to identify painful lumbar segments with the subject’s muscles relaxed. 2) co-contraction phase: the subjects then raises their feet off the floor. If pain identified in the relaxation phase subsides at the co-contraction phase the test is considered positive. PA glide test: Subjects were lying prone and examiners performs PA glide on the lumbar spinous processes. Lack of segmental hypomobility, is considered a positive test. Examiners: two physical therapists who were certified as Orthopaedic Clinical Specialists Time between inter-rater assessments was at least 15 min	Inter-rater reliability Prone instability test for pain (relaxation phase); k (95% CI) k = 0.41 (0.18, 0.63) Prone instability test for pain (co-contraction phase); k (95% CI) k = 0.71 (0.45, 0.98) PA glide test for hypomobility; k (95% CI) k = − 0.02 (− 0.22, 0.18)
Downey et al., 2003 [21] Australia	Inter-rater reliability (*n* = 60) [*n* = 20/pair]	Non-specific LBP < 7 weeks duration, > 18 yrs. old.	Palpation for the spinal level contributing most to the patients’ LBP symptoms (abnormal end-feel, abnormal quality of resistance to motion, and reproduction of pain, local or referred); patient prone, posterior to anterior pressure applied to spinal process and verbal communication between examiner and patient about reproduction of pain. Examiners: three pairs of manipulative physiotherapists with 7–15 yrs. experience and ≥ 3 yrs. experience after postgraduate qualifications in manipulative physiotherapy. Time between inter-rater assessments unknown.	**Inter-rater reliability** Palpation to locate the spinal level; k (95% CI): Overall: k = 0.37 (0.20, 0.54) Pair 1: k = 0.54 (0.26, 0.82) Pair 2: k = 0.45 (0.18, 0.72) Pair 3: k = 0.23 (0.00, 0.46) Palpation to name the spinal level; k (95% CI lower band): Overall: k = 0.09 (0.00, 0.18) Pair 1: k = 0.41 (0.12, 0.70) Pair 2: k = 0.10 (0.00, 0.20) Pair 3: k = − 0.13 (0.00, 0.26)
Hicks et al., 2003 [23] USA	Inter-rater reliability (*n* = 63) [pair 1 *n* = 20, pair 2 *n* = 28, pair 3 *n* = 15]	Low back pain without radiation of pain past the knee, symptom duration unknown, 20 to 66 yrs. old.	Prone instability test: The subject lies prone on the examination table with their feet on the floor. The examiner performs passive intervertebral motion testing for pain. The subject then lifts their feet off the floor. A positive test is when pain provoked during the first part of the test disappears when the legs are lifted up. Passive intervertebral motion testing: with the subject lying prone the examiner applied PA pressure with their hypothenar eminence on each lumbar spinous process. Segmental mobility is judged as normal mobility, hypomobility (more motion than normally expected) and hypermobility (less motion than normally expected). Pain provocation is judged as manual pressure producing pain or not producing pain. Examiners were 4 physical therapists with a least 2 yrs. experience. Examiners were placed in 3 separate pairs. Time between inter-rater assessments was at least 15 min	**Inter-rater reliability** Prone instability test; k (95% CI): k = 0.87 (0.80, 0.94) Pair 1 (*n* = 20): k = 1.0 (1.0–1.0) Pair 2 (*n* = 28): k = 0.81 (0.80–0.94) Passive intervertebral motion tests; k (95% CI): Segmental mobility (dichotomous): Hypermobility k = 0.30 (0.13, 0.47) Hypomobility k = 0.18 (0.05–0.32) Segmental mobility (hypo/normal/hyper): L1 k = 0.26 (− 0.01, 0.53) L2 k = 0.17 (− 0.13, 0.47) L3 k = − 0.02 (− 0.25, 0.28) L4 k = 0.11 (− 0.26, 0.35) L5 k = 0.18 (− 0.03, 0.49) Pain provocation (positive/negative): L1 k = 0.36 (0.12, 0.59) L2 k = 0.45 (0.26, 0.63) L3 k = 0.30 (0.12, 0.47) L4 k = 0.25 (0.11, 0.40) L5 k = 0.55 (0.43, 0.67)
Ravenna et al., 2011 [25] USA	Inter-rater reliability (*n* = 30)	Chronic and recurrent LBP, 18 to 60 yrs. Old.	Prone Instability Test: patient prone with legs over the edge and feet resting comfortably on the floor. The examiner palpates for pain. The patient then raises their legs off the floor and examiner palpates again for pain. A positive test is when pain provoked during the first part of the test disappears or decrease when the legs are lifted up. Examiners were a second-year Doctor of Physical Therapy student and a licensed physical therapist with two years clinical experience. Time between inter-rater assessments was 20 min.	**Prone Instability Test; k (95% CI), PABAK (95% CI)** 0.10 (− 0.27, 0.47), 0.27 (− 0.08, 0.61)
Schneider et al., 2008 [26] USA	Inter-rater reliability (*n* = 39)	Low back pain, symptom duration unknown, 18–65 yrs. old.	Palpation for lumbar segmental mobility, pain provocation and prone instability: patient prone with 1) Prone mobility testing: posterior to anterior joint springing palpation by examiners of SIJs, all lumbar spinous processes and all lumbar facet joints bilaterally; normal or restricted mobility was noted; 2) prone pain provocation testing: patient notifies pain or discomfort provoked while repeating prone mobility test; 3) prone instability test: patient prone with legs over the edge and feet resting comfortably on the floor. The examiner palpates for pain. The patient then raises their legs off the floor and examiner palpates again for pain. A positive test is when pain provoked during the first part of the test disappears when the legs are lifted up. Examiners: two doctors of chiropractic with 25 and 10 years of clinical experience. All the examinations performed in one day.	**Prone Mobility Testing; k (95%CI); PABAK** Left L4–5, and L5-S1 facet mobility: k = − 0.17 (− 0.41,.06); PABAK = 0.08 Right L4–5, and L5-S1 facet mobility: k = − 0.12 (− 0.41,0.18); PABAK = − 0.09 Spinous L4–5 and S1 mobility: k = − 0.05 (− 0.36,0.27); PABAK = 0.11 Left L1–4 facet mobility: k = 0.17 (− 0.14,0.48); PABAK = 0.44 Right L1–4 facet mobility: k = − 0.01 (− 0.33,0.30); PABAK = 0.44 Spinous L1–4 mobility: k = 0.02 (− 0.27,0.32) PABAK = 0.07 Sacroiliac mobility L: k = − 0.11 (− 0.21,-0.01); PABAK = 0.54 Sacroiliac mobility R: k = − 0.10 (− 0.18,-0.02); PABAK = 0.64 **Prone Pain Provocation Testing; k (95%CI): PABAK** Left L4–5, and L5-S1 pain: k = 0.73 (0.51,0.95); PABAK = 0.74 Right L4–5, and L5-S1 pain: k = 0.52 (0.25,0.79); PABAK = 0.54 Spinous L4–5 and L5-S1 pain: k = 0.57 (0.32,0.83); PABAK = 0.58 Left L1–4 pain: k = 0.46 (0.17,0.75); PABAK = 0.48 Right L1–4 pain: k = 0.38 (0.06,0.69); PABAK = 0.54 Spinous L1–4 pain: k = 0.21 (− 0.10,0.53); PABAK = 0.34 Right Sacroiliac pain: k = 0.14 (− 0.19,0.47); PABAK = 0.38 Left Sacroiliac pain: k = 0.33 (0.0,0.66); PABAK = 0.54 **Prone Instability Test; k (95%CI). PABAK** Test 1: k = 0.54 (0.27,0.81); PABAK = 0.58 Test 2: k = 0.46 (0.15,0.77); PABAK = 0.58
Weiner et al., 2006 [29] USA	Inter-rater reliability (*n* = 30)	Chronic LBP, ≥3 months duration, ≥60 yrs. old.	Palpation of the SI joints and lumbar spinous processes to identify pain: 1) SI joints: patient standing on floor with shoes removed, examiner standing behind patient exerts firm pressure over sacroiliac joint, palpation of right joint with right thumb while standing to left side of patient; 2) lumbar spinous processes: examiner behind patient, firmly palpate spinous processes L1–L5 using dominant thumb The examiners underwent training in the protocol with an expert physical therapists to refine and standardize the physical examination procedures Time between intra-rater assessments < 5 min	**SI palpation; k (95% CI not reported)** k = 0.59 **Lumbar spinous palpation** k = 0.47
***Motion Joint Palpation (n = 49)***				
Arab et al., 2009 [20] Iran	Intra-rater and inter-rater reliability (*n* = 25)	LBP around posterior superior iliac spine and buttock, symptom duration unknown, 20–65 yrs. old.	Standing flexion test: The subject is standing and the examiner palpates the movement of PSIS as the subject bends forward. Sitting flexion test: The subject is sitting and the examiner palpates the movement of PSIS as the subject bends forward. Gillett test: The subject is standing with the examiner palpating the movement of PSIS as the subject raises that knee toward their chest. Examiner: two physiotherapists with 1 year experience. Time between intra-rater assessments: 15 min.	**Inter-rater reliability; k (95% CI). PABAK** Standing flexion test R- k = 0.51 (0.08–0.95); PABAK = 0.68 L- k = 0.55 (0.2–0.9); PABAK = 0.60 Sitting flexion test R- k = 0.75 (0.42–1.08); PABAK = 0.84 L- k = 0.64 (0.32–0.96); PABAK = 0.68 Gillet test R- k = 0.41 (0.03–0.87); PABAK = 0.60 L- k = 0.34 (− 0.06–0.7); PABAK = 0.44 **Intra-rater reliability; k (95% CI). PABAK** Standing flexion test Rater 1: R- k = 0.68 (0.35–1.01); PABAK = 0.76 Rater 1: L- k = 0.61 (0.27–0.96); PABAK = 0.68 Rater 2: R- k = 0.60 (0.18–1.02); PABAK = 0.76 Rater 2: L- k = 0.51 (0.08–0.95); PABAK = 0.68 Sitting flexion test Rater 1: k = 0.73 (0.45–1.01); PABAK = 0.76 Rater 1: k = 0.65 (0.34–0.96); PABAK = 0.68 Rater 2: k = 0.65 (0.29–1.02); PABAK = 0.76 Rater 2: k = 0.56 (0.21–0.90); PABAK = 0.60 Gillett test Rater 1: k = 0.42 (− 0.01–0.87); PABAK = 0.60 Rater 1: k = 0.49 (0.09–0.89); PABAK = 0.60 Rater 2: k = 0.25 (− 0.20–0.77); PABAK = 0.52 Rater 2: k = 0.23 (− 0.02–0.67); PABAK = 0.36
Tong et al., 2006 [27] USA	Inter-rater reliability (*n* = 24)	LBP, symptom duration unknown, 32 to 81 yrs. old.	Seated flexion test: the evaluator palpates the cephalad movement at PSISs. As the subject bends forward, the evaluator’s thumbs follow the motion of the PSIS cephalad Standing stork test: the evaluator’s thumb palpates the unilateral movement of left PSIS, and the other thumb palpates the midline of the sacrum. The subject then flexes the left hip and knee to a minimum of 90 degrees. The same is repeated on the right PSIS with the subject flexing the right hip. Standing flexion test: the evaluator palpates the movement of unilateral PSIS. As the subject bends forward to touch the floor, the evaluator’s thumbs follow the PSIS cephalad. The test is repeated on each side. Sacral base position: the subject is sitting, the evaluator palpates the sacral base with the subject’s trunk forward flexed and backward flexed. A positive test is when one side of the sacrum is more anterior or posterior when compared to the other side of the sacrum on the spine motions. Examiners: four physicians. Time between inter-rater assessments unknown.	**k (95% CI not reported); p value** **Seated flexion test:** k = 0.06; *p* = 0.68 **Standing stork test:** k = 0.27; *p* = 0.07 **Standing flexion test:** k = 0.14; *p* = 0.37 **Sacral base position:** flexion k = 0.37; *p* = 0.002 extension k = 0.05; *p* = 0.74
***Static Soft Tissue Palpation (n = 150)***				
Hebert et al., 2015 [22] USA	Inter-rater reliability (n = 32)	Low back pain with ≥20/100 on modified ODI, median duration of symptoms = 205 days, 18 to 60 yrs. old.	Multifidus lift test: to identify lumbar multifidus contraction; participants prone and contralateral arm lifted with/without a hand weight while multifidus muscle palpated immediately lateral and adjacent to the interspinous space of L4–L5 and L5–S1. Examiners: > 10 yrs. clinical experience and approximately 5 yrs. research experience. Time between intra-rater assessments unknown.	**Inter-rater reliability;** k (95% CI) L4–L5 no weight: k = 0.75 (0.52,0.97) L4–L5 weight: k = .0.79 (0.57, 1.00) L5-S1 no weight: k = 0.81 (0.62, 1.00) L5-S1 weight: k = 0.80 (0.59, 1.00)
Jensen et al., 2013 [24] Denmark	Intrarater and inter-rater reliability (*n* = 43)	LBP with or without radiculopathy, variable duration of symptoms, 16–60 yrs. old.	Palpation of gluteal tender points: patient seated, tender points tested from right to left with 4 kg digital pressure on upper outer quadrants of buttocks. Examiners: two consultants in rheumatology and rehabilitation. 20 min between inter-rater assessments and 7 days between intra-rater assessments.	**Intra-rater reliability (95% CI not reported)** Rater A: R k = 0.78; L k = 0.69 Rater B: R k = 0.79; L k = 059. **Inter-rater reliability** Day 1: R k = 0.68; L k = 0.53 Day 2: R k = 0.51; L k = 0.50
Walsh et al., 2009 [28] Ireland	Inter-rater reliability (*n* = 45)	Unilateral low-back related leg pain, mean duration of symptoms = 5.6 months, 18–70 yrs. old.	Palpation of sciatic nerve: With the patient lying prone they are asked if there is any pain or discomfort when the examiner applies gentle pressure at the sciatic nerve bilaterally at the midway point of a line from ischial tuberosity to the greater trochanter of the femur. Examiner: two physiotherapists (eleven yrs. experience with a Masters in Manipulative Therapy and three months clinical experience, respectively).	**Inter-rater reliability sciatic palpation: k (95% CI)** k = 0.80 (0.39–0.94)
Weiner et al., 2006 [29] USA	Inter-rater reliability (*n* = 30)	Chronic LBP, ≥3 months duration, ≥60 yrs. old.	Palpation of the lumbar paraspinal muscles, and piriformis muscles to identify pain:; 1) paralumbar muscles: patient standing on floor with shoes removed, examiner stands behind to left side of patient and braces patient in front with left arm; palpate full extent of right paravertebral musculature with right thumb. Exert approximately 4 kgf. Repeated on the other side 2) piriformis: patient supine flexes right hip and knee, keeping sole of foot on table. Cross bent leg over opposite leg and again place sole on table and exert mild medially directed pressure on lateral aspect of knee to put piriformis in stretch. Exert firm pressure (4 kg) over middle extent of piriformis. Repeated on the other side. The examiners underwent training in the protocol with an expert physical therapists to refine and standardize the physical examination procedures Time between intra-rater assessments < 5 min	**Lumbar paraspinal palpation k (95% CI not reported)** k = 0.34 **Piriformis palpation** k = 0.66

*CI* Confidence interval, *k* Cohen’s kappa, *LBP* Low back pain, *ODI* Oswestry Disability Index, *PA* Posterior to anterior, *PABAK* Prevalence-adjusted and bias-adjusted kappa, *PSIS* Posterior superior iliac spine, *SE* Standard error, *SIJ* Sacroiliac joint, *yrs.* years

**Table 4:** Evidence table for low risk of bias studies assessing the validity of manual palpation tests in patient with low back pain

Authors Year Country	Design^**a**^ Sample size (n)	Population	Index test	Gold/Reference Standard	Validity
***Static Joint Palpation (n = 182)***					
Koppenhaver et al., 2014 [31] USA	Phase II (*n* = 51)	LBP with modified ODI ≥20/100, median duration of symptoms = 184 days, 18–60 yrs. old.	Palpation of spinal stiffness: the spinous processes of L1-L5 palpated with the subject lying prone. The participant was asked to relax as a posterior to anterior (PA) force was applied. Examiner: 1 clinician with 8 yrs. experience	Spinal stiffness was quantified using a mechanized indentation device.	**Criterion validity (95%CI)** Spec: 0.45 (0.28–0.62) Sens: 0.38 (0.21–0.59) +LR: 0.69 (0.37–1.31) -LR: 1.38 (0.82–2.33)
Weiner et al., 2006 [29] USA	Phase I (*n* = 131)	Chronic LBP, mean symptom duration = 158.4 months, ≥60 yrs. old. (*n* = 111) Healthy controls: pain-free individuals (*n* = 20)	Palpation of the SI joints and, lumbar spinous processes to identify pain:1) SI joints: patient standing on floor with shoes removed, examiner standing behind patient exerts firm pressure over sacroiliac joint, palpation of right joint with right thumb while standing to left side of patient, repeated on the other side; 2) lumbar spinous processes: examiner behind patient, firmly palpate spinous processes L1–L5 using dominant thumb; The examiners underwent training in the protocol with an expert physical therapist to refine and standardize the physical examination procedures.	N/A	**Difference between groups: Positive palpation n(%), (*p*value)** **SI palpation** 70 (59), *p* < 0.001 **Lumbar spinous palpation** 59 (53.2), *p* < 0.001
***Motion Joint Palpation (n = 50)***					
Soleimanifar et al., 2017 [32] Iran	Phase II (*n* = 50)	Lumbopelvic pain of unspecified duration,20–65 yrs. old.	Gillet test: the subjects stands while the examiner sits behind the patient and palpates each of the patient’s PSIS, one at a time, with one thumb on the inferior aspect of the PSIS while palpating the sacrum with the other thumb. The subject stands on one leg while pulling the opposite leg up toward the chest. A positive test is if the PSIS on the side of the knee flexion does not move or moves posterior-inferiorly only minimally or even paradoxically moves superiorly Standing flexion test: the subject stands while the examiner sits behind the patient and palpates both of the patient’s |PSIS on their inferior margins. The subject bends forward. A positive result in a standing flexion test indicates limited movement of the ilium on the sacrum. Sitting flexion test: the subject sits while the examiner sits behind the patient and palpates both of the patient’s |PSIS on their inferior margins. The subject bends forward. A positive result indicates limited movement of the sacrum on the ilium. Examiner: one physical Therapist	Thigh thrust test: with the subject lying supine the examiner flexes the hip joint to 90 degree of flexion and slight adduction with the knee flexed. The examiner with one hand cups the sacrum and wraps the other arm and hand around the flexed knee applying axial pressure. A test is positive when pain is provoked over the posterior aspect of the symptomatic SI joint. Faber test: The patient lies supine on the table. The examiner brings the ipsilateral hip into flexion, abduction and external rotation. The foot is rested on unaffected knee. A positive test is when buttock or groin pain below L5 is reproduced Resisted abduction test: The subject supine with the leg fully extended as well as being abducted to 30°. The examiner holds the ankle and pushes medially while the subject pushes laterally. The test is positive when familiar pain is produced over the SIJ below L5.	**Gillet Test:** No validity statistics reported **Standing flexion test:** No validity statistics reported **Sitting flexion test:** No validity statistics reported
***Static Soft Tissue Palpation (n = 545)***					
Adelmanesh et al., 2016 [30] Canada	Phase III (*n* = 337)	LBP with or without radiculopathy of any duration, > 18 yrs. old.	Palpation of the superior-lateral quadrant of the gluteal muscle to identify GTrP representing the combination of tenderness, taut band and pain: With the patient prone the gluteal muscle was compressed with a flat thumb or index finger against the underlying tissue or bone. Examiner: 1 physician with 7 yrs. experience.	Multidisciplinary panel of experts based on examination of clinical evaluations, MRI, and if needed, electrodiagnostic testing. Examiner: 1) clinical evaluation by 1 clinician with 12 yrs. experience; 2) MRI by 1 experienced neuroradiologist and 1 physiatrist; 3) electrodiagnostic testing by a physiatrist with 15 yrs. experience.	Spec (95% CI): 91.4% (86.8–96) Sens (95% CI): 74.1% (67.7–80.3) +LR 8.62 -LR 0.28 PPV (95% CI): 91.9% (87.6–96.3) NPV (95% CI): 72.7% (66.1–79.3) ROC curve (95% CI): 0.827 (0.781–0.874)
Hebert et al., 2015 [22] USA	Phase II (*n* = 32)	Low back pain with ≥20/100 on modified ODI, median duration of symptoms = 205 days, 18 to 60 yrs. old.	Multifidis lift test: to identify lumbar multifidus contraction; participants prone and contralateral arm lifted with/without a hand weight while multifidus muscle palpated immediately lateral and adjacent to the interspinous space of L4–L5 and L5–S1. Examiner: 2 examiners with > 10 yrs. clinical experience and approximately 5 yrs. research experience.	Lumbar multifidus muscle thickness measures at the L4–L5 and L5–S1 spinal levels, at rest and submaximal contraction during contralateral arm lift using brightness-mode real-time ultrasound imaging. Examiner: 1 clinician with 5 years ultrasound experience.	Changes in lumbar multifidus thickness at L4-L5 (*r* biserial correlation coefficient); *p* value. Examiner 1 L4–L5 no weight *r* 0.59; *p* = 0.010 L4–L5 weight *r* 0.71; *p* = 0.003 L5–S1 no weight *r* 0.73; *p* = 0.002 L5–S1 weight *r* 0.62; *p* = 0.008 Examiner 2 L4–L5 no weight *r* 0.71; p = 0.002 L4–L5 weight *r* 0.69; *p* = 0.005 L5–S1 no weight *r* 0.69; p = 0.003 L5–S1 weight *r* 0.63; *p* = 0.009 Changes in lumbar multifidus thickness at L5-S1 (*r* biserial correlation coefficient); p value. Examiner 1 L4–L5 no weight *r* 0.29; *p* = 0.201 L4–L5 weight *r* 0.44; *p* = 0.063 L5–S1 no weight *r* 0.47; *p* = 0.040 L5–S1 weight *r* 0.39; *p* = 0.097 Examiner 2 L4–L5 no weight *r* 0.45; *p* = 0.053 L4–L5 weight *r* 0.24; *p* = 0.0341 L5–S1 no weight *r* 0.44; *p* = 0.056 L5–S1 weight *r* 0.17; *p* = 0.472
Walsh et al., 2009 [28] Ireland	Phase II (*n* = 45)	Unilateral low-back related leg pain, mean duration of symptoms = 5.6 months, 18–70 yrs. old.	Palpation of sciatic nerve: patient prone lying is asked for any pain or discomfort when examiner applies gentle pressure at the sciatic nerve bilaterally at the midway point of a line from ischial tuberosity to the greater trochanter of the femur. Examiner: two physiotherapists (eleven yrs. experience with a Masters in Manipulative Therapy and three months clinical experience, respectively).	Straight leg raise (SLR) and slump tests conducted by one physiotherapist with one year experience.	**Criterion Validity (reference tests: SLR and slump tests) (95% CI)** Spec 0.60 (0.46–0.74) Sens 0.85 (0.75–0.95) PPV 0.63 (0.49–0.77) NPV 0.83 (0.72–0.94) +LR 2.25 -LR 0.25
Weiner et al., 2006 [29] USA	Phase I (*n* = 131)	Chronic LBP, mean symptom duratio*n* = 158.4 months, ≥60 yrs. old. (*n* = 111) Healthy controls: pain-free individuals (*n* = 20)	Palpation of the lumbar paraspinal muscles, and piriformis muscles to identify pain: 1) paralumbar muscles: patient standing on floor with shoes removed, examiner stands behind to left side of patient and braces patient in front with left arm; palpate full extent of right paravertebral musculature with right thumb. Exert approximately 4 kgf: 2) piriformis: patient supine flexes right hip and knee, keeping sole of foot on table. Cross bent leg over opposite leg and again place sole on table and exert mild medially directed pressure on lateral aspect of knee to put piriformis in stretch. Exert firm pressure (4 kg) over middle extent of piriformis, The examiners underwent training in the protocol with an expert physical therapist to refine and standardize the physical examination procedures.	N/A	**Difference between groups: Positive palpation n(%), p value** **Lumbar paraspinal palpation** 60(50), *p* < 0.001 **Piriformis palpation** 57(51.4), *p* < 0.001

*GTrP* Gluteal trigger point, *LBP* Low back pain, *+LR* Positive likelihood ration, *−LR* Negative likelihood ration, *N/A* Not Applicable, *NPV* Negative predictive value, *ODI* Oswestry Disability Index, *PPT* Pressure pain threshold, *PPV* Positive predictive value, *Sens* Sensitivity, *SI* Sacroiliac, *Spec* Specificity, *yrs.* years

^**a**^: Design refers to Phase I-IV questions as described by Sackett & Haynes (2002). 2002;324(7336):539–41

No arbitrary classification was used to report the strength of reliability or validity findings. Such classification used arbitrary cut-points that do not take into account the level of misclassification that can be acceptable in specific context. Rather, values of kappa coefficients, sensitivity, specificity etc. were reported. The authors interpreted the kappa and measurement errors according to clinical settings and purposes of palpation tests in their context. Kappa scores of < 0.6 are considered to have no, minimal or weak agreement and kappa scores of > 0.6 are considered to have moderate, strong or almost perfect agreement [38]. This should be used as a rough guide when interpreting the kappa and measurement errors according to clinical settings and purposes of palpation tests in individual context.

### Statistical analyses

We computed kappa coefficients (k) and 95% confidence intervals (CI) to determine the inter-rater reliability of our screening methodology of articles. We computed the percentage agreement between reviewers for the classification of articles into high or low risk of bias.

### Reporting

This review complies with the Reporting Items for Systematic Reviews and Meta-Analyses (PRISMA) statement (Additional file 4) [39]. The Statement for Reporting Studies of Diagnostic Accuracy (STARD) was used to inform in the critical appraisal with the QAREL and QUADAS-2 [40].

## Results

### Study selection

**Fig. 1:**
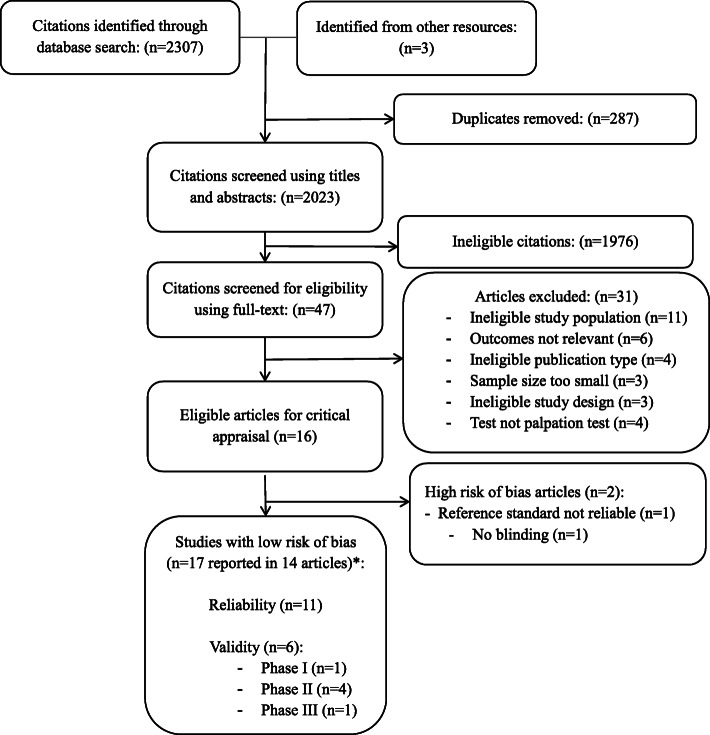
Identification and selection of articles on reliability and validity of manual palpation used to assess patients with low back pain. *Not mutually exclusive

We identified 2307 citations (plus 3 citations from other resources) removed 287 duplicates, and reviewed 2023 articles for eligibility (Fig. 1). In stage 1 screening, 1976 citations were ineligible. Forty-seven papers were reviewed in stage 2, and 31 were excluded: ineligible study population (*n* = 11) [41–51], inappropriate outcome measure (*n* = 6) [52–57], ineligible publication type (*n* = 4) [58–61], ineligible sample size (*n* = 3) [62–64], study design (*n* = 3) [65–67] and did not investigate manual palpation (*n* = 4) [68–71]. Two authors were contacted for publication type and age range, both responded [27, 59].

We critically appraised 16 articles and 14 articles had low risk of bias and were included in our evidence synthesis [19–32] (Fig. 1). Over the 16 articles appraised, 14 articles including 17 studies were reported (three articles included both reliability and validity in their study). The inter-rater agreement for screening of articles was Kappa = 0.86 (95% CI 0.73–0.98). The percentage agreement for the admissibility of studies was 100% (17 agreements/17 studies over the 16 articles appraised).

### Study characteristics

Fourteen articles had a low risk of bias [19–32]. Of those, 11 reported on the reliability of palpation tests [19–29] and six reported on validity [22, 28–32]. Three articles examined both reliability and validity [22, 28, 29].

The eleven reliability studies with low risk of bias examined inter-rater reliability of manual palpation to assess joints mobility or motion [19–21, 23, 26, 27], pain [19, 21, 23–26, 28, 29] and muscle contraction [22]. Two of the eleven studies also examined intra-rater reliability of manual palpation assessing joint motion [20] and muscle tenderness [24]. The six validity studies included one phase I study on palpation of joints and muscles to assess pain [29], four phase II on palpation of nerves to elicit pain [28], spinal stiffness [31] muscle contraction [22] and sacroiliac joint motion [32] and one phase III study on palpation of gluteal muscle for tenderness and pain [30].

The 14 low risk of bias articles investigated: 1) static joint palpation (*n* = 7) [19, 21, 23, 25, 26, 29, 31], 2) motion joint palpation (*n* = 3) [20, 27, 32], and 3) static soft tissue palpation (*n* = 5) [22, 24, 28–30] (Tables 3 and 4). They assessed various techniques: 1) joint pain provocation [19, 21, 23, 26, 29], 2) pain or tenderness of muscles [24, 29, 30], 3) pain and tenderness of nerves [28], 4) joint stiffness/mobility [19, 21, 23, 25, 26, 31], 5) joint motion [20, 27, 32], and 6) isometric muscle contraction [22]. Table 5 showed a glossary of definitions for all of the palpation tests included in the articles.

**Table 5:** Glossary of Manual Palpation Tests in Accepted Articles

Test	Purpose of Test	Description of Test
***Static Joint Palpation***		
Lumbar spinous palpation Weiner et al., 2006 [29]	Palpation to identify pain	The examiner is behind patient and firmly palpates the spinous processes of L1–L5 using their dominant thumb. A positive test is pain on palpation.
Passive intervertebral motion tests Hicks et al. 2003 [23]	Lumbar palpation for segmental mobility and pain	With the subject lying prone the examiner applies AP pressure with their hypothenar eminence on each lumbar spinous process. Segmental mobility is judged as normal, hypomobile and hypermobile. Pain provocation is judged as pressure producing pain or not producing pain.
Posterior/Anterior Glide Test Alyazedi et al. 2015 [19]	Palpation for identify lumbar spinal mobility.	Subjects are lying prone and examiners performs PA glide on the lumbar spinous processes. Lack of segmental hypomobility, is considered a positive test.
Prone Instability Test Alyazedi et al. 2015 [19] Hicks et al. 2003 [23] Ravenna et al., 2011 [25] Schneider et al., 2008 [26]	Lumbar springing palpation for pain	Patient prone with legs over the edge of table and feet resting comfortably on the floor. The examiner palpates for pain. The patient then raises their legs off the floor and examiner again palpates for pain. A positive test is indicated by painful segments in the first position becoming nonpainful with contraction of the back extensors.
Prone Lumbar Palpation (comparable level and level identification) Downey et al., 2003 [21]	Identification of spinal level most contributing to LBP and identification of that level	Palpation for the spinal level contributing most to the patients’ LBP symptoms (abnormal end-feel, abnormal quality of resistance to motion, and reproduction of pain, local or referred); patient prone, posterior to anterior pressure applied to spinal process and verbal communication between examiner and patient about reproduction of pain.
Prone Mobility Test Schneider et al., 2008 [26]	Lumbar segmental mobility	Posterior to anterior joint springing palpation by examiners of SIJs, all lumbar spinous processes and all lumbar facet joints bilaterally. A positive test is the palpation of restricted motion.
Prone Pain Provocation Test Schneider et al., 2008 [26]	Lumbar spine pain	Patient notifies the examiner of pain or discomfort provoked while repeating prone mobility test
Sacroiliac Joint Palpation Weiner et al., 2006 [29]	Palpation of the sacroiliac joints for pain	The patient stands on floor with shoes removed and the examiner stands behind patient. The examiner exerts firm pressure over sacroiliac joint, palpation of right joint with right thumb while standing to left side of patient and palpation of the left joint with the left thumb while standing to the right of the patient. A positive test is the patient reporting pain in the back.
Spinous Palpation for Stiffness Koppenhaver et al., 2014 [31]	Joint springing of the lumbar spinous process	The spinous processes of L1-L5 are palpated with the subject lying prone. The participant was asked to relax as a posterior to anterior (PA) force was applied. Each vertebral segment was judged to be hypermobile, hypomobile or normal mobility.
***Motion Joint Palpation***		
Gillet Test Arab et al., 2009 [20] Soleimanifar et al., 2017 [32]	Palpation for movement at the PSIS while patient raises knee	The subject is standing with the examiner palpating the PSIS as the subject raises that knee toward their chest. A positive test is when the PSIS on the side of the knee flexion does not move or moves posterior-inferiorly only minimally or even paradoxically moves superiorly.
Sacral Base Position Test Tong et al., 2006 [27]	Palpation of the sacral base for position while the patient flexes then extends their spine	The subject is sitting, the evaluator palpates the sacral base with the subject’s trunk forward flexed and backward flexed. A positive test is when one side of the sacrum is more anterior or posterior when compared to the other side of the sacrum on the spine motions.
Seated Flexion Test Tong et al., 2006 [27]	Bilateral palpation for cephalad movement at the PSIS while patient forward bends spine	The evaluator palpates both PSISs. As the subject bends forward, the evaluator’s thumbs follow the motion of the PSIS cephalad. If one side moves more cephalad than the other side by more than 1 cm, the side that moves more is considered abnormal.
Sitting Flexion Test Arab et al., 2009 [20] Soleimanifar et al., 2017 [32]	Palpation for movement at the PSIS while patient forward bends spine	The subject is sitting and the examiner palpates the PSIS as the subject bends forward. A positive result in this test indicates limited movement of the sacrum on the ilium.
Standing Flexion Test Arab et al., 2009 [20] Soleimanifar et al., 2017 [32] Tong et al., 2006 [27]	Palpation for movement at the PSIS while patient forward bends spine	The subject is standing and the examiner palpates the PSIS as the subject bends forward. A positive result in a standing flexion test indicates limited movement of the ilium on the sacrum.
Standing Stork Test Tong et al., 2006 [27]	Palpation for movement at the PSIS while patient raises knee	The evaluator’s thumb palpates the unilateral PSIS, and the other thumb palpates the midline of the sacrum. The subject then flexes the left hip and knee to a minimum of 90 degrees. The same is repeated on the right PSIS with the subject flexing the right hip. The sacroiliac joint motion is considered normal if the thumb on the PSISs moves caudal and abnormal if the thumb on the PSISs does not move or if it rises.
***Static Soft Tissue Palpation***		
Palpation of Gluteal Muscle Jensen et al., 2013 [24]	Gluteal muscle palpation for tenderness	Patient seated, tender points tested from right to left with 4 kg digital pressure on upper outer quadrants of buttocks. A positive test was pain on palpation.
Multifidus Lift Test Hebert et al., 2015 [22]	Palpation of the multifidus muscle for contraction while patient raises while lifting contralateral arm	Participants prone and contralateral arm lifted with/without a hand weight while multifidus muscle palpated immediately lateral and adjacent to the interspinous space of L4–L5 and L5–S1. A test was judged as normal or abnormal lumbar multifidus contraction.
Palpation of Gluteal Muscle Adelmanesh et al., 2016 [30]	Palpation of the superior-lateral quadrant of the gluteal muscle to identify GTrP.	Palpation of the superior-lateral quadrant of the gluteal muscle to identify GTrP representing the combination of tenderness, taut band and pain: With the patient prone the gluteal muscle was compressed with a flat thumb or index finger against the underlying tissue or bone. The points were considered GTrP when the combination of taut band, tenderness, and pain recognition were present.
Palpation of Lumbar Paraspinal Muscles Weiner et al., 2006 [29]	Lumbar paraspinal muscle palpation for pain	With the patient standing on the floor with shoes removed, the examiner stands behind to left side of patient and braces patient in front with left arm; palpate full extent of right paravertebral with right thumb. Exert approximately 4 kgf. The test is repeated on the other side. A positive test is when the patient reports pain when the muscle is palpated.
Palpation of Piriformis Muscle Weiner et al., 2006 [29]	Piriformis muscle palpation for pain	With the patient supine they flex their right hip and knee, keeping sole of foot on table. The bent leg is crossed over opposite leg and again the sole of the foot is placed on table. Mild pressure is exerted medially directed on the lateral aspect of knee to put piriformis in stretch. Exert firm pressure (4 kg) over middle extent of piriformis. The test is repeated on the other side. A positive test is when the patient reports pain when the muscle is palpated.
Palpation of Sciatic Nerve Walsh et al., 2009 [28]	Sciatic nerve palpation for pain	With the patient lying prone they are asked if there is any pain or discomfort when the examiner applies gentle pressure at the sciatic nerve bilaterally at the midway point of a line from ischial tuberosity to the greater trochanter of the femur. A positive test is pain or discomfort over the sciatic nerve.

The duration of LBP varied across studies: < 7 weeks (1/14 articles) [21], > 4 weeks (1/14 articles) [24], ≥ l months (1/14 articles) [29], new episode to > 3 months (1/14 articles) [19] and unspecified duration (10/14 articles) [20, 22, 23, 25–28, 30–32]. The studies were conducted in Australia [21], Canada [30], Denmark [24], Iran [20, 32], Ireland [28], and the United States [19, 22, 23, 25–27, 29, 31] between 2003 and 2017.

We did not perform a meta-analysis because of the heterogeneity of studies in symptom duration, palpation technique, and outcome specification.

### Assessment of risk of Bias

Tables 1 and 2 showed the risk of bias for scientifically admissible reliability and validity studies based on the modified QAREL and QUADAS-2 criteria respectively.

The low risk of bias studies met the following criteria: 1) clearly described objective; 2) representative sample; 3) representative raters; 4) blinding of the test results between raters; 5) appropriate and valid standard test; and 6) appropriate statistical analysis (Tables 1 and 2). However, these studies had the following limitations: 1) unclear time interval between tests (*n* = 1) [27]; 2) no blinding for intra-examiner reliability (*n* = 2) [20, 24]; 3) 30 min rest period between the repeat testing between the same examiner and no blinding to clinical information (*n* = 2) [27, 28]; 4) unclear blinding to clinical information or additional clues [23]; 5) no blinding to clinical information and unclear blinding to additional clues (*n*=8) [21, 22, 23, 24, 25, 27, 28, 29] and 6) non-random or unclear administration of tests (*n* = 5) [19, 23, 27–29]. Most validity studies had appropriate exclusion criteria and blinding. However, validity studies had limitations: 1) four studies did not use a consecutive or random sample [22, 29, 31, 32]; and 2) two studies were unclear as to whether an appropriate time interval between tests were used [28, 31]; 3) one study was unclear as to whether an appropriate reference standard (slump test and straight leg raise) was used [28]; 4) in one study the examiner was not blinded to the results of the index or reference test [32] and 5) in one study it was unclear as to whether all patients were included in the analysis [22].

Two validity studies were excluded after critical appraisal. Abbott et al. used flexion/extension radiographs as a reference standard without establishing the test-retest reliability of patient positioning when taking of the radiographs [72]. Telli et al. didn’t use blinding in their reliability study [73].

### Summary of evidence

#### Reliability of joint and bony structure palpation

##### Static palpation

Four studies investigated static palpation to elicit pain. Overall, these studies suggest that important measurement error is associated with eliciting pain from: 1) lumbar facet joints (inter-rater reliability 0.38 ≤ k ≤ 0.73); 2) lumbar spinous processes (inter-rater reliability 0.21 ≤ k ≤ 0.57); 3) sacro-iliac (SI) joints (inter-rater reliability 0.14 ≤ k ≤ 0.59) [19, 23, 26, 29] (Table 3). Similarly, the evidence suggests that static palpation used to identify joint segmental mobility has low inter-rater reliability (i.e., lumbar facet joints: − 0.17 ≤ k *≤* 0.17; and lumbar spinous processes; − 0.02 *≤* k *≤* 0.26 SI joints: − 0.11 ≤ k ≤ − 0.10) [19, 23, 26]. The inter-rater reliability of the prone instability test for pain ranged from a kappa of 0.30 [23], 0.41 [19] and 0.54 [26] in the relaxation phase of the test and a kappa of 0.46 [26], 0.71 [19] and 0.87 [23] in the contraction phase of the test. In a study that combined the two phases of the test into a positive or negative finding reported a kappa of 0.10 [25] (Table 3). Furthermore, a third study by Downey et al. (2003) reported low inter-rater reliability of joint static palpation to locate the spinal level (0.23 ≤ k ≤ 0.54) and name the spinal level (− 0.13 ≤ k ≤ 0.41) in patients with LBP symptoms [21] (Table 3).

##### Motion palpation

We found inconsistent evidence in support of the reliability of motion palpation of the lumbar spine and SI joints to assess joint motion [20, 27]. The inter-rater reliability of motion palpation of the sacroiliac joint varied (inter-rater reliability 0.14 ≤ k ≤ 0.75 and intra-rater reliability 0.23 ≤ k ≤ 0.73) (Table 3) [20, 27]. Tong et al. (2006) suggested that sacral position cannot be reliably assessed during trunk motion using sacral base position test (inter-rater reliability: flexion k = 0.37, extension k = 0.05) [27].

#### Reliability of soft tissue palpation

##### Static palpation

We found varying levels of reliability for the palpation of the soft tissue structures associated with low back pain [22, 24, 28, 29]. The inter-rater reliability ranged from k = 0.80 for sciatic nerve pain, to 0.51 ≤ k ≤ 0.68 for gluteal tender points and k = 0.34 for lumbar paraspinal muscle pain [24, 28, 29]. One study suggested that the multifidus muscle can be reliably assessed by examiners who believe they are palpating the multifidus muscle for abnormal isometric contraction by palpating lateral and adjacent to the interspinous space of L4-L5 and L5-S1 with contralateral arm raising both with and without using hand weights (inter-rater reliability 0.75 ≤ k ≤ 0.81) [22]. It is possible that the multifidus lift test is also palpating a more superficial muscle which raises questions about the validity of this test.

#### Validity of joint and bony structure palpation

##### Static palpation

Two studies investigated the validity of static joint palpation [29, 31]. One phase I study found that pain elicited by palpation of the SI joints and lumbar spinous processes was more common in LBP patients compared to healthy controls [29]. One phase II study reported that posterior to anterior palpation used to identify stiffness from L1-L5 had a sensitivity of 38% (95% CI 21–59%), a specificity of 45% (95% CI 28–62%), a positive likelihood ratio of 0.69 (95% CI 0.37–1.31) and a negative likelihood ratio of 1.38 (95% CI 0.82, 2.33) when compared to a mechanized indentation device [31] (Table 4).

##### Motion palpation

One phase II study investigated the validity of joint motion palpation tests for the sacroiliac joints [32]. They examined the relationship between sacroiliac tests for joint motion (Gillet test, sitting flexion test and standing flexion test) and sacroiliac pain provocation tests (Faber test, thigh thrust test and resisted abduction test) but did not use statistics for validity (Table 4).

#### Validity of soft tissue palpation

##### Static palpation

Four studies investigated the validity of static soft tissue palpation [22, 28–30]. One phase I study found that pain elicited by palpation of the lumbar paraspinal and piriformis muscles was more common in LBP patients compared to without LBP [29]. A phase II study tested the validity of the multifidus lift test with and without hand weights to identify abnormal isometric multifidus muscle contraction when compared to measurement with real-time ultrasound imaging of lumbar multifidus muscle thickness [22] (Table 4). The authors reported that the multifidus lift test correlates with ultrasound finding at the L4–5 level (*r* biserial correlation coefficient: 0.59 without hand weight and 0.73 without hand weight) and weakly associated at the L5-S1 level (*r* biserial correlation coefficient: 0.17 and 0.47) (Table 4) [31]. Another phase II study investigated the validity of sciatic nerve palpation between the ischial tuberosity and the greater trochanter for pain using the straight leg raise and slump test as reference standard to evaluate mechanosensitivity of the sciatic nerve [28]. The authors found that sciatic nerve palpation had a sensitivity of 85% (95% CI, 75–95%) and a specificity of 60% (95% CI, 46–74%) [26]. Finally, one phase III study investigated the validity of static palpation of gluteal muscle for taut band, tenderness and pain recognition compared to an expert panel confirmation of radicular LBP (informed by MRI and electro-diagnostic testing). The authors reported that static palpation of the gluteal muscle had a sensitivity of 74.1% (95% CI, 67.7–80.3%) and a specificity of 91.4% (95% CI, 86.8–96.0%) in identifying radicular pain [30].

## Discussion

### Summary of results

We reviewed the reliability and validity of manual palpation used to assess patients with LBP. We retrieved eleven studies on the reliability of static and motion palpation of joint and soft tissue. Overall, the evidence suggest that static joint palpation is not reliable in identifying pain and segmental mobility of the lumbar facet joints, lumbar spinous processes and SI joints, and location of spinal level contributing LBP symptoms. However, static soft tissue palpation may help reliably identify gluteal tender points, sciatic nerve pain, and multifidus contraction but not lumbar paraspinal muscle pain. We identified six validity studies for the assessment of LBP using static joint, joint motion and soft tissue palpation. Gluteal muscle palpation for pain was able to help identify differentiate LBP patients with or without radiculopathy (phase III study). We found preliminary evidence for the validity of the piriformis and lumbar paraspinal muscle palpation for pain (phase I study), spinous and sacroiliac joint palpation for pain (phase I study), sciatic nerve palpation for pain to identify mechanosensitivity of the sciatic nerve as determined by the straight leg raise and slump test (phase II study) and the multifidus lift test to help identify abnormal isometric contraction (phase II study); and against posterior to anterior palpation used to identify stiffness from L1-L5 spine levels (phase II study). Sacroiliac joint motion tests were not associated with sacroiliac pain provocation tests (phase II study). Overall, very little knowledge is available to support the usefulness of palpation of the lumbar and sacroiliac test when examining patient with low back pain.

### Comparison with previous systematic reviews

The results of our systematic review differ from previous systematic reviews [9, 11, 13]. Our finding that static joint palpation of the spinous processes, facet and sacroiliac joints is not reliable to identify pain disagrees with previous systematic reviews [9, 11, 13]. Three reviews reported that the reliability of static joint palpation for pain was acceptable, but the kappa used to make this conclusion is low (k ≥ 0.4) [9, 11, 13]. Our review disagrees with the previous finding by Stochkendahl et al. et al. that found that static soft tissue palpation may help reliably identify soft tissue pain (k ≤ 0.4) [11]. Our review found inconsistent reliability to identify soft tissue pain with the inclusion of three recent studies [22, 24, 28]. The different conclusions may be due to different search strategies, new evidence, inclusion of small sample studies, use of self-developed checklists, or use of predefined cut-off points to differentiate low and high quality studies in the four systematic reviews. However, our results are consistent with a systematic review published in 2020 focusing only on segmental motion palpation [74]. Poor evidence regarding reliability and validity of segmental motion testing were reported and clinical use of stand-alone tests cannot be recommended [74].

### Strengths and limitations

Our systematic review has several strengths. First, our comprehensive search strategy of multiple databases was developed by a health sciences librarian in consultation with content experts and was then reviewed by an independent health sciences librarian using the PRESS Checklist [18]. Second, we used detailed, predefined inclusion and exclusion criteria to capture a diffuse range of possibly relevant citations. Third, we used paired independent reviewers to screen and critically appraise citations to minimize bias and error. The critical appraisal was completed by trained reviewers using standardized quality assessment tools (QAREL/QUADAS-2). Fourth, bias in reported results was minimized by performing a best-evidence synthesis that included only high-quality studies. Finally, we only included studies that tested subjects with LBP. This makes our results more generalizable to the patients seen by practitioners in clinical practice.

Our review also had limitations. First, our search was limited to studies published in English and French languages. It is possible that relevant studies in other languages may have been excluded. Second, our search may not have retrieved all relevant studies, although our search strategy was comprehensive and the search was conducted in multiple major medical databases. Third, our search was limited to studies published after 2000. Fourth, it is possible that individual differences in scientific judgment could have resulted in varied critical appraisal outcomes among reviewers. This bias was minimized using training with the standardized assessment tools and a consensus process for determining internal validity of studies. Finally, studies examining motion palpation tests had smaller sample sizes (validity studies *n* = 50; reliability studies *n* = 49) than studies of static joint or muscle palpation. This may have limited the precision of the results and led to uncertainty in our assessment of motion palpation tests.

### Clinical implications

Our review found very little evidence for the use of manual palpation to assess low back pain patients. Manual palpation tests suffered from misclassification error in that they were unable to differentiate those with LBP to subjects without LBP. Soft tissue palpation of the sciatic nerve, gluteal muscles for pain and the multifidus muscle for isometric contraction were reliable but have not been tested sufficiently for their validity for use in clinical practice. Although we did find that gluteal muscle palpation of trigger points and taut bands is valid to differentiate LBP patients with or without radiculopathy in a clinical setting. We found very limited evidence to support the use of joint palpation and clinician should reconsider its diagnostic value when assessing patients with low back pain.

## Conclusion

We synthesize the evidence on the reliability and validity of manual palpation to assess adults with LBP. The evidence does not support reliability of joint palpation but static soft tissue palpation is reliable. There is little evidence on the motion joint palpation used in LBP patients. Gluteal muscle palpation for pain was able to differentiate LBP patients with or without radiculopathy (phase III study). We found preliminary evidence from Phases I and II validity studies for some palpation tests. High quality phase III and IV validity studies are required to understand the diagnostic value of manual palpation tests in the assessment of adults with LBP. Clinicians must reconsider the usefulness of these tests when examining patients.

## Supplementary Information

**Additional file 1.**

**Additional file 2.**

**Additional file 3.**

**Additional file 4.**

## Data Availability

Not applicable.
