# Supplementary material for: Reliability and validity of manual palpation for the assessment of patients with low back pain: a systematic and critical review
Source: Chiropr Man Therap. 2021 Aug 26;29:33. doi: 10.1186/s12998-021-00384-3 (PMC8390263; doi:10.1186/s12998-021-00384-3)
Supplement: Supplementary file 1 — Additional file 1. [file 12998_2021_384_MOESM1_ESM.docx]

**Appendix I: Medline Search Strategy**

1. MH "Reproducibility of Results+"
2. MH "Sensitivity and Specificity"
3. MH “Reliability and Validity+”
4. MH "Predictive Value of Tests”
5. MH “False Positive Results”
6. MH “False Negative Results”
7. MH “Likelihood Functions”
8. reproducibility
9. sensitiv*
10. specificity
11. predict* n2 value*
12. reliab*
13. valid*
14. false positive*
15. false negative*
16. accura*
17. roc curve* or received operating characteristic
18. kappa coefficient*
19. intra-rater* or inter-rater* or interrater* or intrarater* or rater* or intra-examiner* or inter-examiner* or intraexaminer* or interexaminer*
20. utility n2 test*
21. likelihood ratio*
22. likelihood function*
23. MH "Odds Ratio"
24. odds ratio*
25. MH "ROC Curve"
26. test-retest
27. responsive*
28. MH "Diagnosis"
29. MH "Diagnostic Techniques and Procedures"
30. MH "Diagnostic Self Evaluation"
31. diagnos* n2 (technique* or procedure* or evaluat*)
32. assess*
33. evaluat*
34. exam*
35. procedure*
36. screen*
37. or/1-36
38. exp Back/
39. exp Back Injuries/
40. exp Back Pain/
41. Coccyx/in [Injuries]
42. Intervertebral Disc Degeneration/
43. Intervertebral Disc Displacement/
44. Lumbar Vertebrae/in [Injuries]
45. exp Lumbosacral Plexus/
46. Lumbosacral Region/in [Injuries]
47. Osteoarthritis, Spine/
48. Piriformis Muscle Syndrome/
49. Polyradiculopathy/
50. Sacrococcygeal Region/
51. Sacroiliac Joint/
52. Sacrum/
53. Sciatica/
54. Spinal Diseases/
55. Spinal Stenosis/
56. (avulsed lumbar adj3 (disc* or disk*)).ab,ti.
57. (back adj3 (ache* or injur* or pain*)).ab,ti.
58. (backache* adj3 (injur* or pain*)).ab,ti.
59. (back pain or back-pain).ab,ti.
60. coccydynia.ab,ti.
61. coccyx.ab,ti.
62. dorsalgia.ab,ti.
63. (lumbar disc* adj3 (extruded or degenerat* or herniat* or prolapse* or sequestered or slipped)).ab,ti.
64. (lumbar disk* adj3 (extruded or degenerat* or herniat* or prolapse* or sequestered or slipped)).ab,ti.
65. "low back pain" or lower back pain or low-back pain.ab,ti.
66. "low back-pain*" or lower back-pain.ab,ti.
67. (lumbar adj3 (pain or facet or nerve root* or osteoarthritis or radicul* or spinal stenosis or spondylo* or zygapophys*)).ab,ti.
68. "lumbarsacr*".ab,ti.
69. lumboischialgia.ab,ti.
70. "lumbosacr*".ab,ti.
71. "Piriformis syndrome*".ab,ti.
72. radiculalgia.ab,ti.
73. (sacral adj2 pain*).ab,ti.
74. (sacrococcygeal adj2 pain*).ab,ti.
75. (sacroiliac or sacro-iliac).ab,ti.
76. "sciatic*".ab,ti.
77. (SI adj joint).ab,ti.
78. (spinal adj stenos?s).ab,ti.
79. spondylosis.ab,ti.
80. "tailbone adj3 pain*".ab,ti.
81. "vertebrogenic adj3 pain*".ab,ti.
82. or/38-81
83. MH “Palpation”
84. MH “Pain Threshold”
85. MH “Pressure”
86. MH “Trigger Points”
87. palpat*
88. pain threshold*
89. trigger point*
90. tender point*
91. tenderness
92. pressure n2 (sensitiv* or threshold*)
93. stiffness
94. passive assessment
95. joint* n2 restrict*
96. joint* n2 fixat*
97. subluxation
98. algomet*
99. manual exam*
100. manual assess*
101. OR/83-100
102. AND/36, 82, 101
103. Limits ENGLISH, FRENCH
104. Limits Jan 2000-July 11, 2019
