# Supplementary material for: Reliability and validity of manual palpation for the assessment of patients with low back pain: a systematic and critical review
Source: Chiropr Man Therap. 2021 Aug 26;29:33. doi: 10.1186/s12998-021-00384-3 (PMC8390263; doi:10.1186/s12998-021-00384-3)

**Appendix II:** Quality Appraisal Tool for Studies of Diagnostic Reliability (QAREL) criteria for diagnostic reliability studies (Lucas et al., 2010).


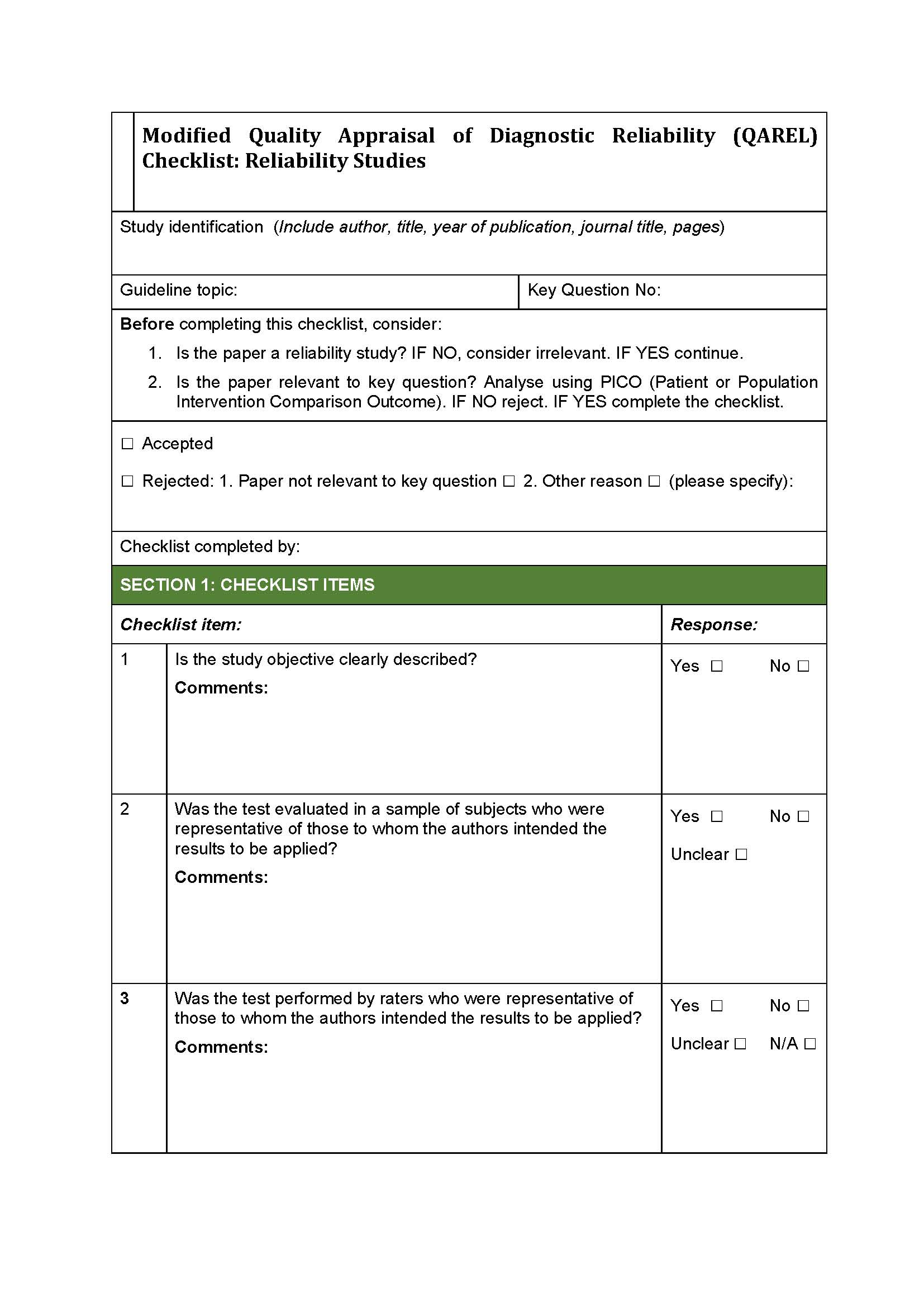


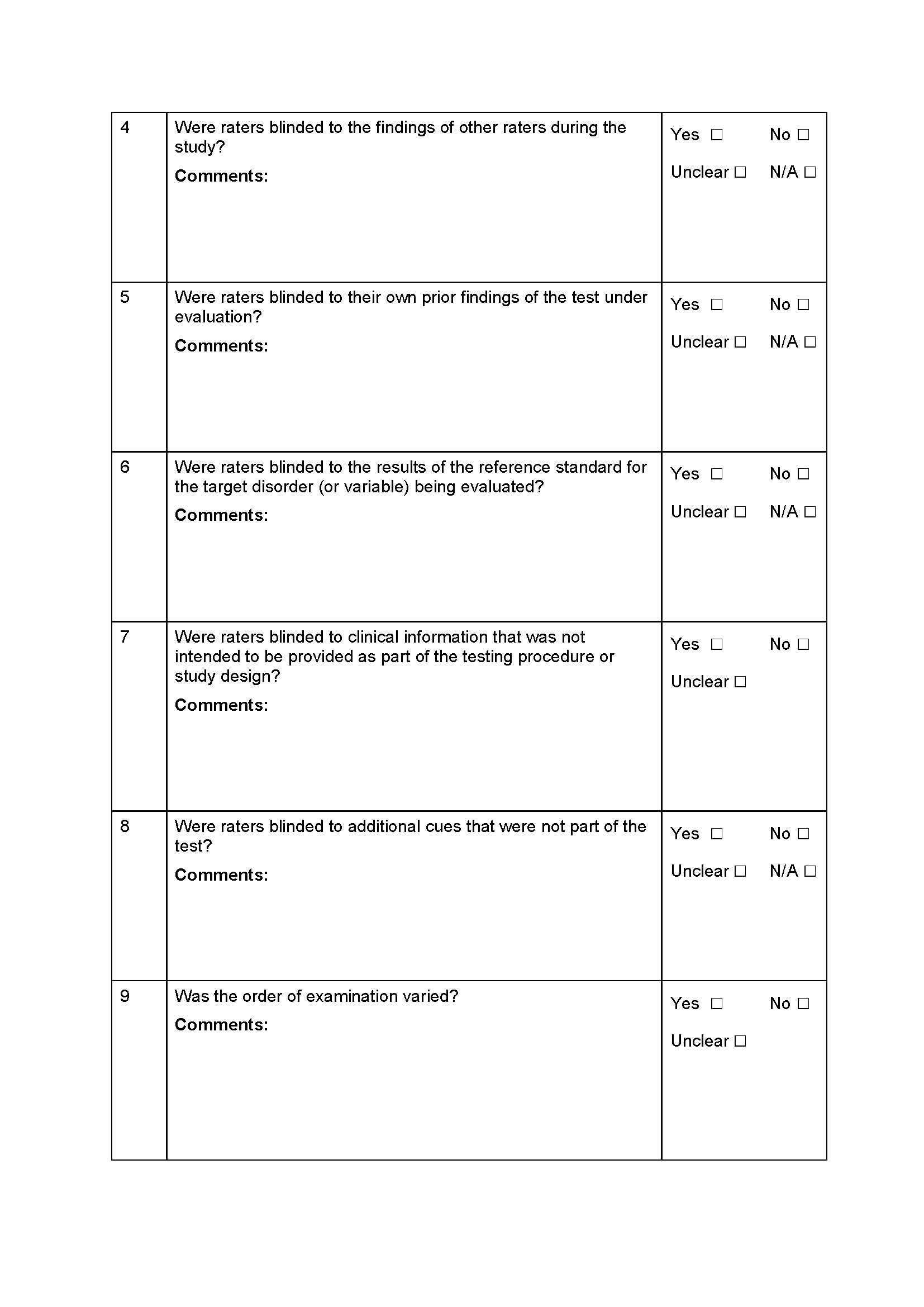


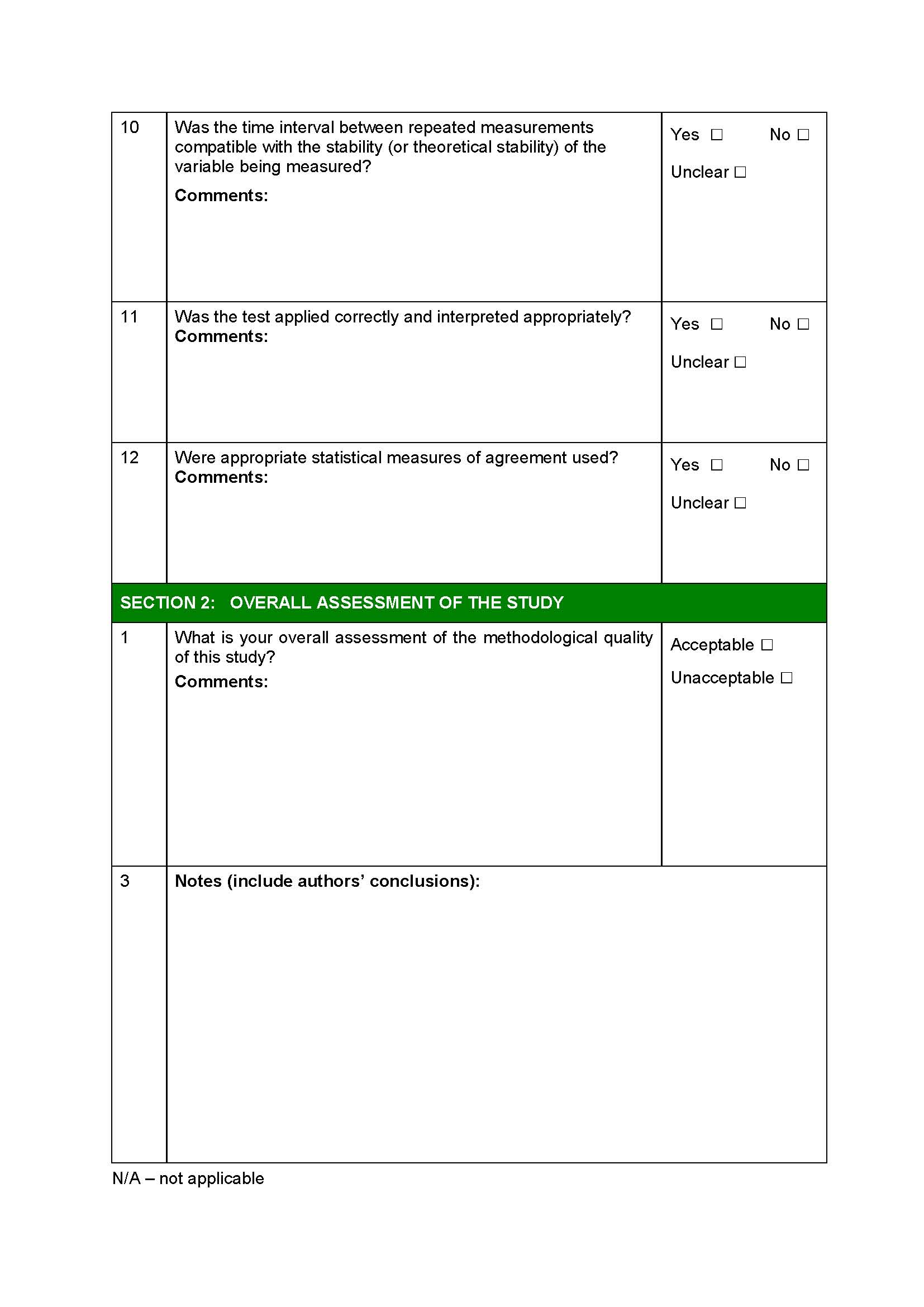

Supplement: Supplementary file 2 — Additional file 2. [file 12998_2021_384_MOESM2_ESM.docx]
