# Supplementary material for: Reliability and validity of manual palpation for the assessment of patients with low back pain: a systematic and critical review
Source: Chiropr Man Therap. 2021 Aug 26;29:33. doi: 10.1186/s12998-021-00384-3 (PMC8390263; doi:10.1186/s12998-021-00384-3)
Supplement: Supplementary file 3 — Additional file 3. [file 12998_2021_384_MOESM3_ESM.docx]

**Appendix III:** Modified Quality Assessment of Diagnostic Accuracy Studies-2 (QUADAS-2) criteria for diagnostic accuracy studies (Lucas et al., 2010).

**
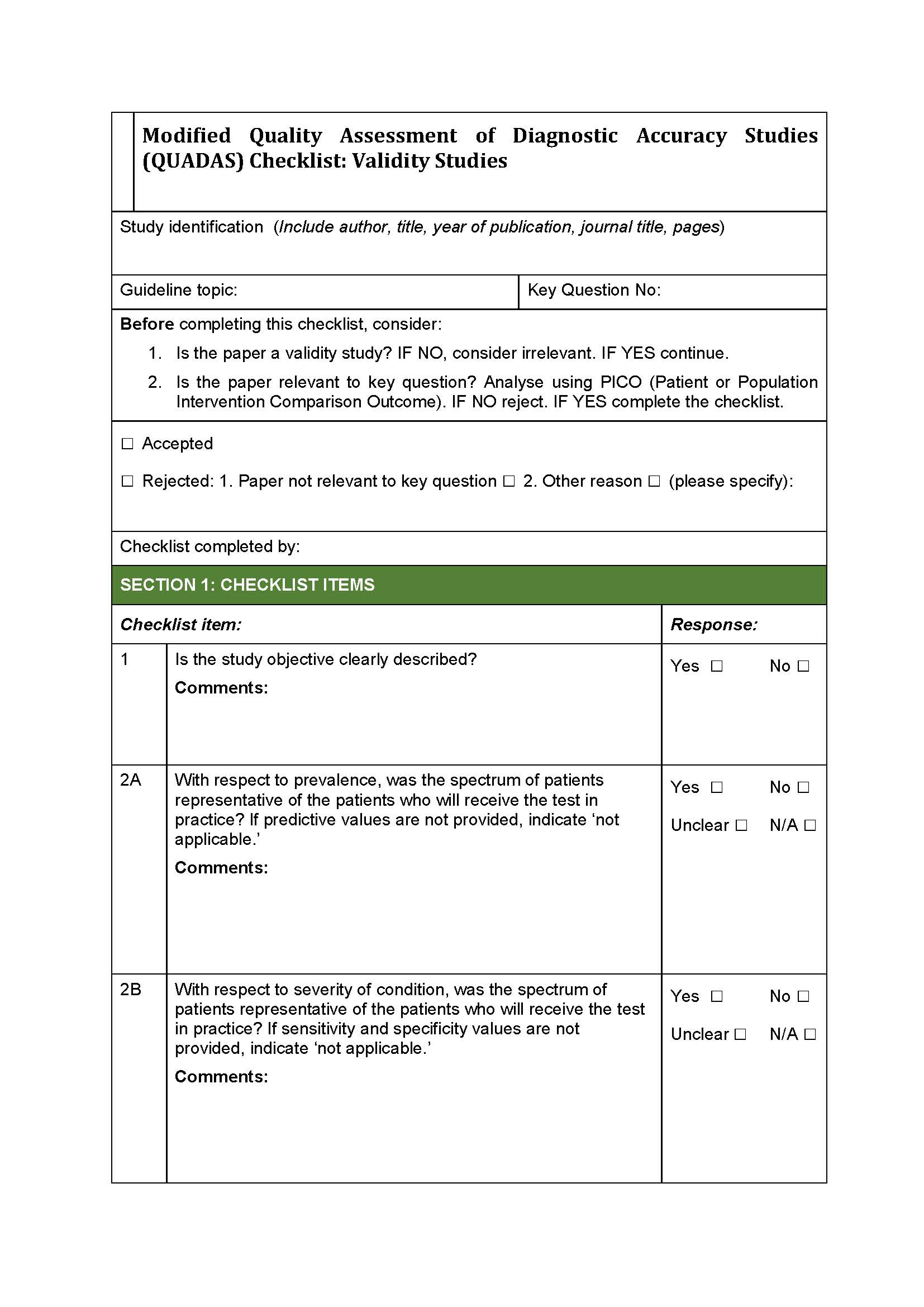
**

**
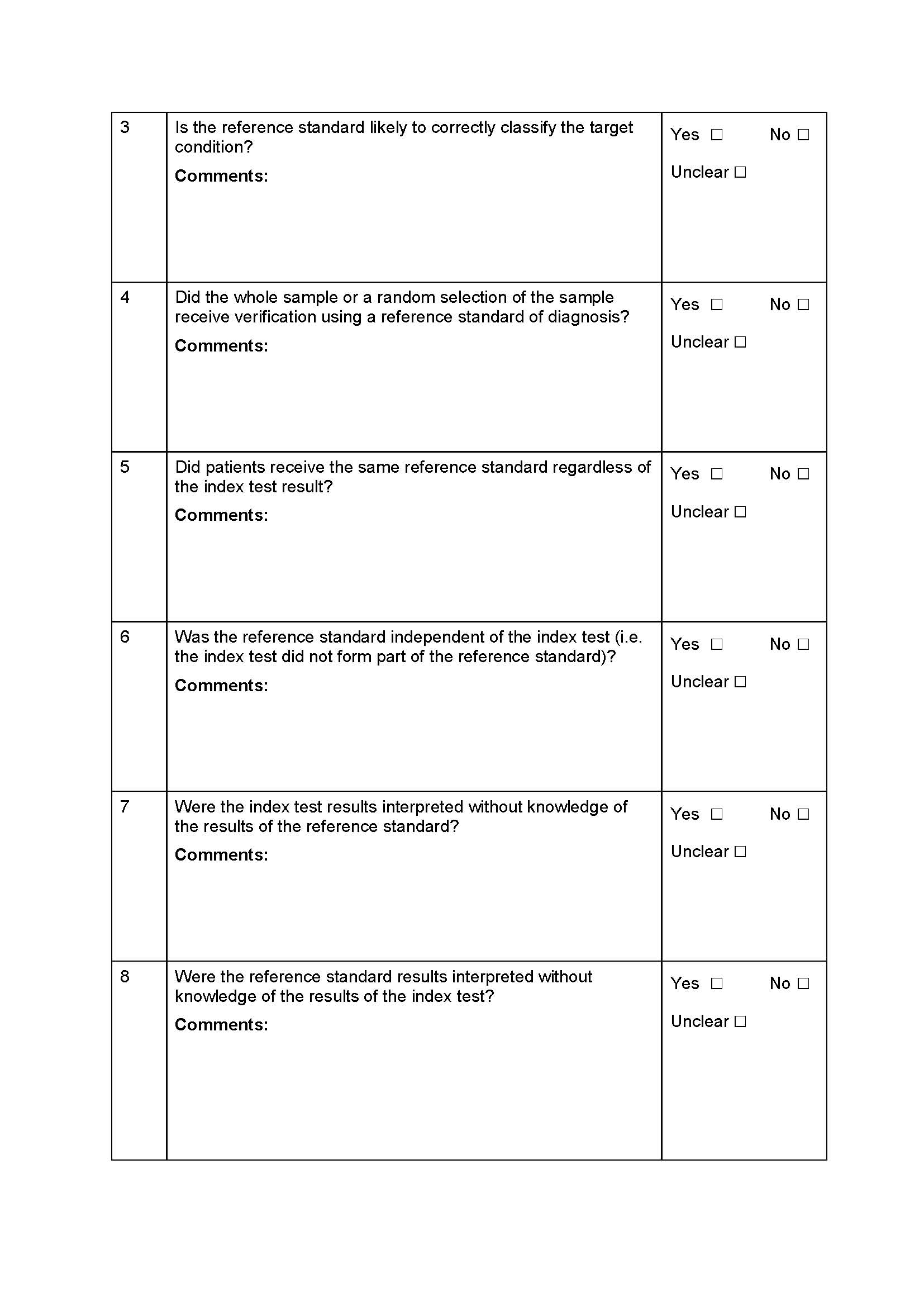
**

**
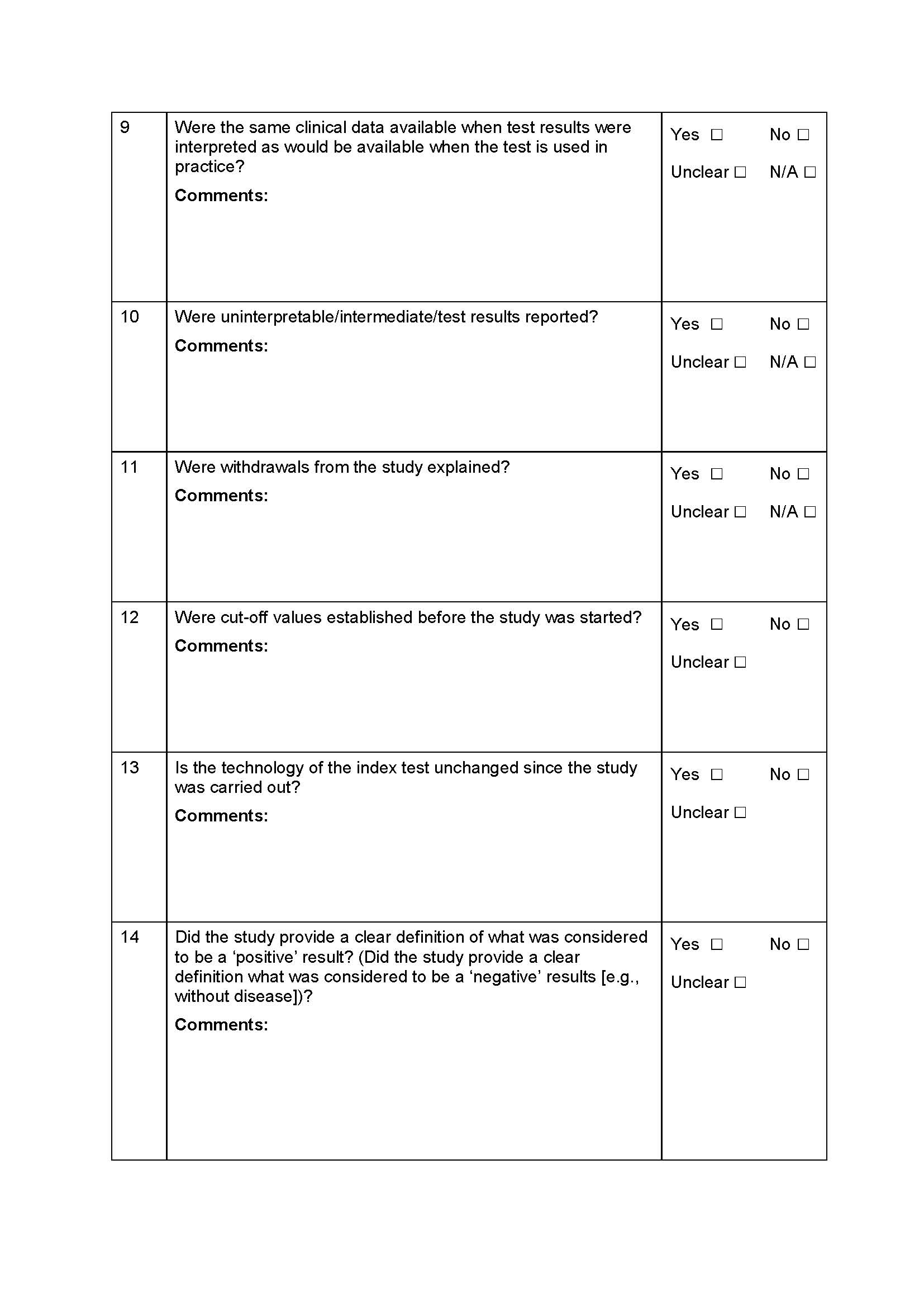
**

**
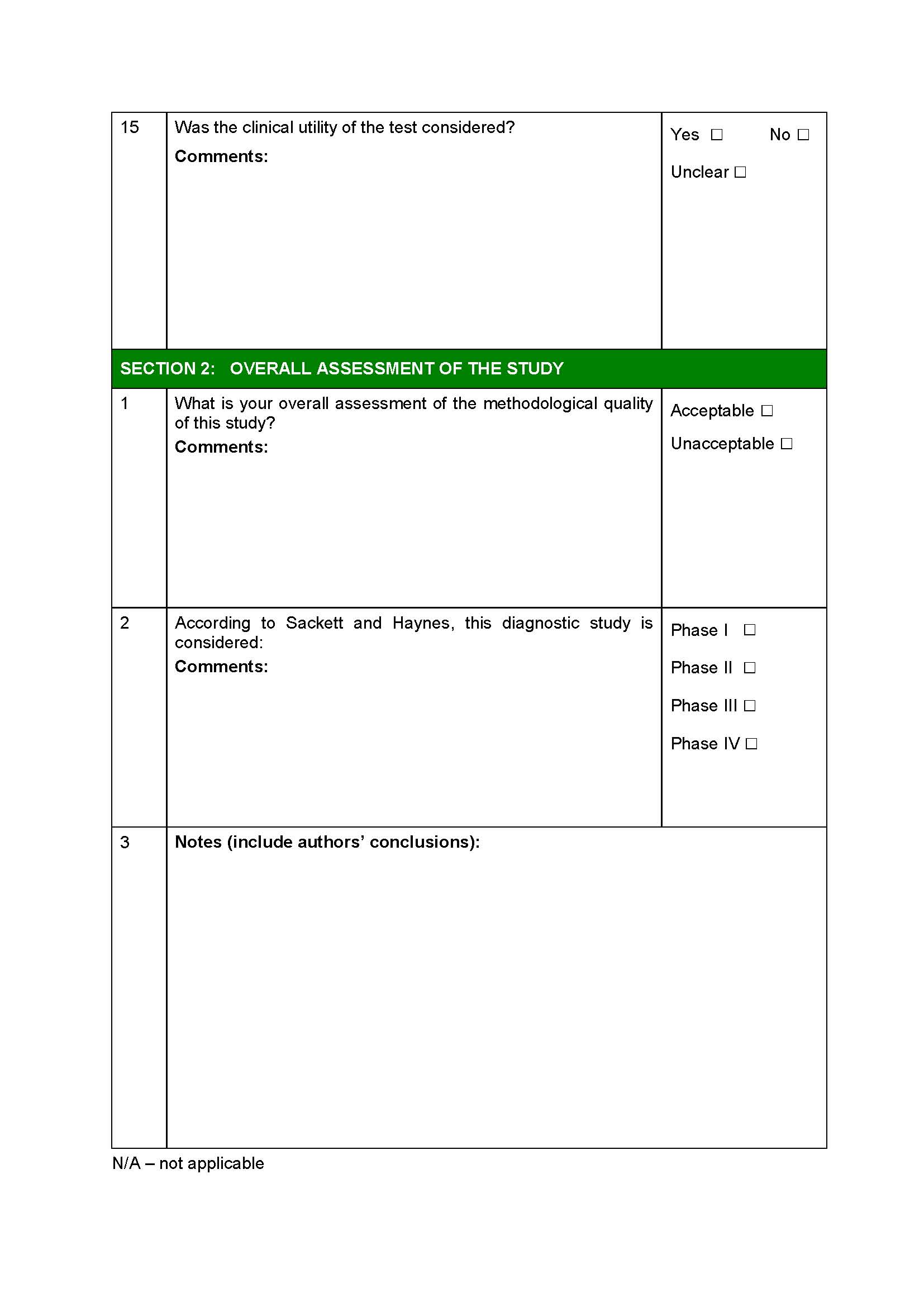
**
